# Transformer's frequency response analysis results interpretation using a novel cross entropy based methodology

**DOI:** 10.1038/s41598-023-33606-0

**Published:** 2023-04-23

**Authors:** Chander Parkash, Ali Reza Abbasi

**Affiliations:** 1 Department of Mathematics, Rayat Bahra University, Mohali, 140 104 India; 2 Department of Electrical, Faculty of Engineering, Fasa University, Fasa, Fars Iran

**Keywords:** Electrical and electronic engineering, Applied mathematics, Computational science

## Abstract

Transformer defects can be identified by the FRA (frequency response analysis) that is a promising diagnostic technique. Despite the standardization in FRA measuring technique, its results interpretation is yet a research area. Because different faults types can be identified in various frequency bounds of the FRA signatures, it is necessary to identify the possible relationships between specific failures and frequency ranges in this contribution. For this purpose, a real transformer is used to conduct the essential tests, which include both healthy and faulted circumstances (axial displacement (AD), radial deformation (RD), and short-circuits (SC)). To identify efficient characteristics from the produced frequency response traces and improve interpretation accuracy of such traces, a new hyperbolic fuzzy cross entropy (FCE) measure is demonstrated and then utilized for the aim of discrimination and classification of transformer winding defects in pre-defined frequency ranges. After normalizing FRA results of the transformer under healthy and various fault circumstances the lower bounds from such responses have been extracted and then utilized to construct the desired form of the fuzzy sets of healthy and faulted circumstances. Then, a new hyperbolic FCE measure-based discrimination and classification of winding faults methodology is offered on the basis of highest and lowest FCE measure values. The highest FCE measure value between the fuzzy sets of healthy and faulted circumstances such as AD, RD and SC is designated to confirm the occurrence of winding faults in a suitable frequency range. The suggested methodology ensures smart interpretation of FRA signature and accurate classification of winding faults as it can effectively discriminate both healthy and faulted circumstances in the desired frequency ranges. The proposed approaches' performance is tested and compared by applying the experimental data after feature extraction.

## Introduction

Power grid transformers are a necessary but expensive piece of equipment. In the course of their service life, transformers are susceptible to mechanical or electrical changes, such as winding deformation, movement, or turn to turn^1^. In order to prevent catastrophic transformer failures, winding defects must be identified as soon as possible^2^. For the reasons outlined above, transformer operation status condition monitoring has grown in popularity around the world^3^. Many theoretical and practical methods for diagnosing winding electrical and mechanical faults are now being proposed. The FRA method has been employed in recent years to check the condition of transformers. Comparative methods such as the Transfer function (TF) approach can be used to identify any discrepancies between the fingerprint signature and the FRA signature^4^. Winding faults such as axial displacement (AD), short circuits (SC), and radial deformation (RD), are all too common^5^. FRA signature comparison can indicate the location, severity, and type of a failure in a transformer if any of the aforementioned problems occur. As a result, this comparison relies heavily on individual experience rather than established and widely accepted codes. For the time being, the interpretation of results from FRA measures has not been standardized, despite valid criteria being produced^6^. Therefore, a new technique based on fuzzy cross entropy measure for smart interpretation of FRA spectrum has been developed, tested, and assessed in this study work.

FRA's ability to detect faults in transformers is constantly expanding as a result of the growing use of this technology. FRA can now detect a greater number of transformer issues than ever before. FRA signature interpretation has been studied extensively^7–9^, but a trustworthy analysis of the FRA traces remains a difficult research challenge. The concepts of testability analysis and parametric faults are of great importance in the field of fault diagnosis for analog circuits based on FRA. The total number of parameters of testable system is called testability degree. Faults can be classified into parametric faults and catastrophic faults. Parametric faults are examined in this research, especially the parameter values deviation from a certain tolerance range. Diagnostic methods called simulation after test are used for this type of faults. In these methods, element values are identified using input–output relationships and comparison between circuit responses. A set of equations is gained from this comparison. Fault detection equations that consider the real values of the parameters as unknowns are constituted by these equations. In the circuit under test, the testability is provided by the degree of solvability of these equations. Therefore, efforts to isolate undetectable faults are necessary to avoid wasting resources and time. To improve interpretation accuracy of the produced frequency response traces and to identify efficient characteristics form such traces, the reported approaches have been found difficult in achieving the desired objectives. As of now, there are a variety of approaches for interpreting FRAs, including those involving electric model modelling, artificial intelligence, and mathematics. The first way uses several circuit parts to represent each section of the winding^10^. First, the variations in transformer construction are translated into the corresponding modifications in circuit components. As a result, the varying parts are then incorporated into a circuit model for analysis^11^. There are several drawbacks to this method^12^. The fundamental problem of the circuit model is the difficulty in incorporating mechanical failures. To help explain the FRA curves, finite element analysis (FEA) commonly used to generate an analogous electric model of transformer winding^13^. The FRA curve beyond 1 MHz can be studied using Zhang's hybrid model and FEA^14^. Finding a precise model of the winding from the frequency response, on the other hand, remains a difficult challenge.

Using smart classifiers to identify problems falls under the second category^3,5^. These methods extract the frequency response features (primarily the numerical and statistical indicators) that are necessary for testing and training classifiers, and these features are employed for both. For the classification of winding faults by means of artificial intelligence, Bigdeli has used the support vector machine (SVM) technique^3^. Using digital image processing and polar plots^15^, Aljohani et al. have developed a new FRA interpretation method for detecting short-circuit faults of transformer winding as well as radial deformation and bushing defects. Moreover, cross-correlation characteristics and algorithms based upon ANN are being utilized to distinguish electrical & mechanical problems^16^. Several academics have developed methods based on ANN and SVM techniques that necessitate a larger number of incorrect cases in order to train the neurons and store the data^17,18^. As a last resort, numerical and statistical indicators, which are simple and accurate, are frequently used because of this. It has been studied extensively by E. Rahimpour to investigate the winding faults in terms of the amplitude and frequency deviation, weight functions, standard difference area, as well as the other indices^7^. Samimi summarizes the most recent statistics indicators in^19^. Numerical and statistical methods are also encouraged by the IEEE standard. Additional to their ability to work on their own, these indices can also be employed with other algorithms. To yet, no indices have been shown to be particularly effective at analyzing the fault degrees associated with various fault types. For solving this problem, a large number of FRA signatures of various winding defect degrees and types were acquired by artificial fault simulation on a specially designed transformer model that is represented in^20^. However, the statistical indicators approach has room for improvement.

To address these shortcomings, we have proposed a novel hyperbolic fuzzy cross entropy measure-based distinction and classification of transformer winding defects procedure to cluster the FRA results of power transformers under various faulty and healthy conditions and improve interpretation accuracy.^21^ presents a fuzzy set theory that can play a prominent role to improve winding fault diagnosis accuracy under fuzzy environment. However, since Zadeh’s invention of fuzzy sets theory, fuzzy sets have been reconstructed into various forms of other sets including neutrosophic sets with single valued, fuzzy sets with interval valued intuitionistic, intuitionistic fuzzy sets^22^, and so on. Surprisingly, the existing winding faults classification methods have been found deprived of using the fuzzy sets theory. However, the cross-entropy measures based upon the fuzzy sets of normalized frequency responses of a transformer can be developed and deployed for accurate classification of electrical and mechanical winding faults. To ensure smart interpretation of FRA signature and accurate classification of winding faults, an attempt has been accomplished in this pathway by introducing a novel hyperbolic fuzzy cross entropy measure that can discriminate both healthy and faulted circumstances in the desired frequency ranges. The projected hyperbolic fuzzy cross entropy measure based on the fuzzy sets of normalized frequency responses of the transformer is compatible with the existing cross entropy measures due to Shang and Jiang^23^ and Bhandari and Pal^23^.

Below are a few of the most noteworthy features of the suggested FCE measure-based discrimination and classification of winding faults methodology.

1. Normalization of the produced frequency responses of the transformer under healthy and various fault circumstances.
2. Extraction of lower bounds from the normalized frequency responses and their utilization to construct the desired form of fuzzy sets.
3. Computation of FCE measure values between the fuzzy sets of health and faulted circumstances
4. Confirmation of the transformer winding defects includes AD, RD and SC based upon the highest FCE measure values
5. Classification of various transformer winding faults employing the proposed FCE measure-based methodology is introduced for the first time in this study and can be deployed to determine the transformer’s status
6. Because the winding faults of the transformer have a substantial effect on different frequency bands, the proposed approach is separately studied in the high, mid, and low frequency regions.
7. Interpretations of the FRA results can be conveyed both graphically (descriptive statistics) and numerically (inferential statistics).
8. Assisting the operator in making a decision by presenting a tool.
9. Applying the extracted feature to malfunctioning transformers in order to test its reliability.

The next sections are laid out as follows. "Problem description" section explains problem description including the background of frequency response analysis, the essential prerequisites of information theory needed to understand the suggested study, the creation of a new entropy measure as hyperbolic fuzzy followed by another new fuzzy cross entropy based on two sets of symmetric hyperbolic fuzzy in healthy and faulted conditions of a transformer. "Fuzzy cross entropy based distinction and classification of transformer winding defects procedure" section introduces the proposed FCE measure based on the distinction and taxonomy of transformer winding defects procedure. "Case study" section introduces the test case and explains how to recognize and classify various types of faults. "Conclusion" section concludes the paper with a discussion of the findings.

## Problem description

### Background of frequency response

**Figure 1:**
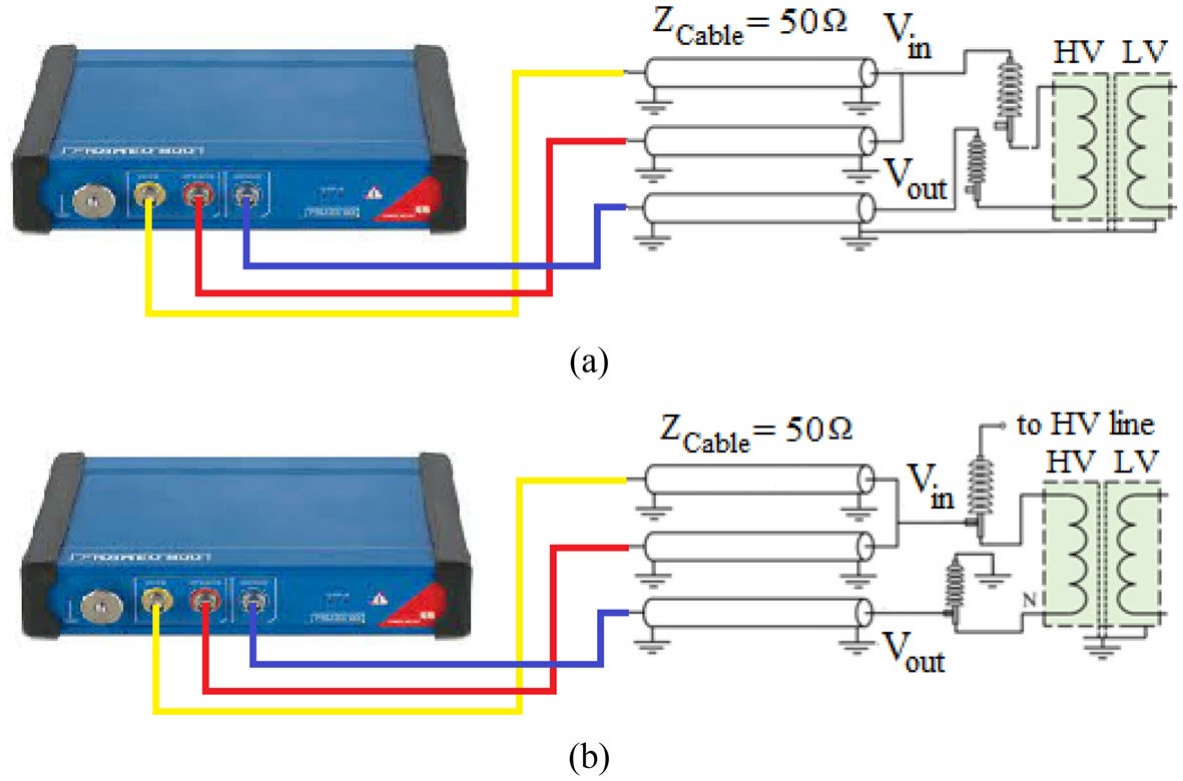
the setup of FRA measurement: (**a**) off-line; (**b**) on-line setup.

FRA is a well-established industrial technique^24^ that uses at the transformer input terminal a sinusoidal reference signal and analyzes the reaction of the winding from its other side when the transformer is out of service (off-line FRA)^24^. Offline FRA measurements are shown in Fig. 1a. Moreover, Fig. 1b indicates on-line FRA setup (while it is in service). In this method, an excitation signal is injected into the tap of the bushing and the response of the winding is checked from the tap of the lateral bushing^25–28^. There are advantages and disadvantages to every test configuration. Frequencies between 20 Hz and 2 MHz are commonly employed in FRA to analyze transformer frequency response signatures, and the mechanical structure of the winding can be studied in this large frequency spectrum. FRA data interpretation complexity is a difficulty for accurate prognostic methods. Visual interpretation of the present FRA spectrum is the most used method today. To perform this, the difference between the measured FRA spectrum and the signature is classified into high, medium, and low frequency ranges, and then the analysis is separately performed on each frequency range. In this manner, the interpreting expert's experience is critical, as is his or her thorough understanding of the effects of each parameter on the FRA spectrum. As a result, the interpretation is more susceptible to errors owing to human mistake because of this technique. Therefore, in the following, fuzzy cross entropy method will be used to cluster FRA data under healthy and faulted situations and improve interpretation accuracy in the following section.

### Underlying Theories

#### Preliminaries

For understanding the fundamental concepts of our proposed methodology, it is necessary to introduce the following definitions.

##### Definition 2.1.

*Fuzzy Set (FS):* A fuzzy set $P_{FS}^{a}$ in a finite discourse of universe $U = \left( {x_{1} ,x_{2} ,...,x_{n} } \right)$ can be represented by the form: $P_{FS}^{a} = \left( { < x_{i} ,\mu_{{P^{a} }} \left( {x_{i} } \right) > |x_{i} \in U} \right)$ where $\mu_{{P^{a} }} \left( {x_{i} } \right):U \in \left[ {0,1} \right]$ refers to as membership function and satisfies $0 \le \mu_{{P^{a} }} (x_{i} ) \le 1$. Also, the complement $C\left( {P_{FS}^{a} } \right)$ of the fuzzy set $P_{FS}^{a} \in U$ is an object represented by $C\left( {P_{FS}^{a} } \right) = \left( {x_{i} ,1 - \mu_{{P^{a} }} (x_{i} ) > |x_{i} \in U} \right)$.

##### Definition 2.2.

*Symmetric Fuzzy Cross Entropy:* Let $P_{FS}^{a} = \left( { < x_{i} ,\mu_{{P^{a} }} \left( {x_{i} } \right) > |x_{i} \in U} \right)$ and $Q_{FS}^{a} = \left( { < x_{i} ,\mu_{{Q^{a} }} \left( {x_{i} } \right) > |x_{i} \in U} \right)$ are any two fuzzy sets in $U = \left( {x_{1} ,x_{2} ,...,x_{n} } \right)$ which are quantified by membership functions $\mu_{{P^{a} }} \left( {x_{i} } \right),\mu_{{Q^{a} }} \left( {x_{i} } \right):U \to \left[ {0,1} \right]$ with the condition $0 \le \mu_{{P^{a} }} \left( {x_{i} } \right),\mu_{{Q^{a} }} \left( {x_{i} } \right) \le 1.$ Then, a function $H_{CE} :F\left( U \right) \times F\left( U \right) \to Rz^{ + }$ is called as symmetric fuzzy cross entropy^29,30^ based on two fuzzy sets $P_{FS}^{a}$ and $Q_{FS}^{a}$ if

$(i)\,H_{CE} \left( {P_{FS}^{a} ,Q_{FS}^{a} } \right) \ge 0\forall P_{FS}^{a} ,Q_{FS}^{a} \in F\left( U \right)$ with the equality sign if $P_{FS}^{a} = Q_{FS}^{a} .$

$(ii)\,H_{CE} \left( {P_{FS}^{a} ,Q_{FS}^{a} } \right) = H_{CE} \left( {Q_{FS}^{a} ,P_{FS}^{a} } \right)$. In other words, $H_{CE} \left( {P_{FS}^{a} ,Q_{FS}^{a} } \right)$ is symmetric in nature.

$(iii)\,H_{CE} \left( {C\left( {P_{FS}^{a} } \right),C\left( {Q_{FS}^{a} } \right)} \right) = H_{CE} \left( {P_{FS}^{a} ,Q_{FS}^{a} } \right)$ which means $H_{CE} \left( {P_{FS}^{a} ,Q_{FS}^{a} } \right)$ does not change whenever $P_{FS}^{a}$ and $Q_{FS}^{a}$ are replaced with their complements.

$(iv)\,H_{CE} \left( {P_{FS}^{a} ,Q_{FS}^{a} } \right)$ should satisfy the convexity property with respect to both the membership functions $\mu_{{P^{a} }} \left( {x_{i} } \right)$ and $\mu_{{Q^{a} }} \left( {x_{i} } \right).$

#### A new hyperbolic fuzzy cross entropy (FCE) measure

To discriminate both healthy and faulted circumstances in the desired frequency ranges, we first establish a novel hyperbolic fuzzy cross entropy measure (Theorem 2.1) as follows.

##### Theorem 2.1.

Let $P_{FS}^{a} = \left( { < x_{i} ,\mu_{{P^{a} }} \left( {x_{i} } \right) > |x_{i} \in U} \right)$ and $Q_{FS}^{a} = \left( { < x_{i} ,\mu_{{Q^{a} }} \left( {x_{i} } \right) > |x_{i} \in U} \right)$ are two fuzzy sets in $U = \left( {x_{1} ,x_{2} ,...,x_{n} } \right).$ Set $T_{0} = \mu_{{P^{a} }} \left( {x_{i} } \right) + \mu_{{Q^{a} }} \left( {x_{i} } \right),T_{1} = \mu_{{p^{a} }}^{2} \left( {x_{i} } \right) + \mu_{{Q^{a} }}^{2} \left( {x_{i} } \right),T_{2} = \sqrt {\mu_{{P^{a} }} \left( {x_{i} } \right)} + \sqrt {\mu_{{Q^{a} }} \left( {x_{i} } \right)} ,T_{3} = \left( {1 - \mu_{{P^{a} }} \left( {x_{i} } \right)} \right)^{2} + \left( {1 - \mu_{{Q^{a} }} \left( {x_{i} } \right)} \right)^{2} ; T_{4} = \sqrt {1 - \mu_{{P^{a} }} \left( {x_{i} } \right)} + \sqrt {1 - \mu_{{Q^{a} }} \left( {x_{i} } \right)} ,T_{5} = \sqrt {\mu_{{P^{a} }} \left( {x_{i} } \right)\mu_{{Q^{a} }} \left( {x_{i} } \right)} .$

Then $\,H_{CE}^{\mu } \left( {P_{FS}^{a} ,Q_{FS}^{a} } \right)$ is a valid symmetric hyperbolic fuzzy cross entropy (Definition 2.2) hinged on two fuzzy sets $P_{FS}^{a}$ and $Q_{FS}^{a}$ defined by

1 $$H_{CE}^{\mu } \left( {P_{FS}^{a} ,Q_{FS}^{a} } \right) = \sum\limits_{i = 1}^{n} {\left[ { - 6\sinh \left( \frac{1}{8} \right) + \left( {2 + T_{0} } \right)\sinh \,\left( {\frac{{1 + T_{1} }}{{8 + T_{2}^{4} }}} \right) + \left( {4 - T_{0} } \right)\sinh \,\left( {\frac{{1 + T_{3} }}{{8 + T_{4}^{4} }}} \right)} \right]} \,\,$$

##### *Proof.*

In view of Definition 2.3, $\,H_{CE}^{\mu } \left( {C\left( {P_{FS}^{a} } \right),C\left( {Q_{FS}^{a} } \right)} \right) = \,\,H_{CE}^{\mu } \left( {P_{FS}^{a} ,Q_{FS}^{a} } \right)$ is straightforward for each $P_{FS}^{a} ,Q_{FS}^{a} \in F\left( U \right).$ Also, the necessary condition (ii) is obvious. Establishment of the following Lemma 2.1 is necessary for the purpose of proving the non-negativity of $\,H_{CE}^{\mu } \left( {P_{FS}^{a} ,Q_{FS}^{a} } \right).$

##### Lemma 2.1.

In our usual notations, there exist the inequality $\sqrt {\frac{{T_{1} }}{2}} \ge \frac{{T_{2}^{2} }}{4}$ with equality if $\mu_{{P^{a} }} \left( {x_{i} } \right) = \mu_{{Q^{a} }} \left( {x_{i} } \right)\forall i = 1,2,...,n.$

##### Proof.

The inequality $\sqrt {\frac{{T_{1} }}{2}} \ge \frac{{T_{2}^{2} }}{4}$ will be satisfied if

2a $$\frac{{T_{1} }}{2} \ge \frac{{T_{2}^{4} }}{16}\;\;{\text{or if}}\;\;8T_{1} \ge T_{2}^{4} .$$

But $$T_{2}^{4} = \left( {\sqrt {\mu_{{P^{a} }} \left( {x_{i} } \right)} + \sqrt {\mu_{{Q^{a} }} \left( {x_{i} } \right)} } \right)^{4} = \left( {\mu_{{P^{a} }} \left( {x_{i} } \right) + \mu_{{Q^{a} }} \left( {x_{i} } \right) + 2\sqrt {\mu_{{P^{a} }} \left( {x_{i} } \right)\mu_{{Q^{a} }} \left( {x_{i} } \right)} } \right)^{2} = \left( {T_{0} + 2T_{5} } \right)^{2} = T_{0}^{2} + 4T_{5}^{2} + 4T_{0} T_{5} .$$

With this simplification, the resulting inequality (2) reduces to

$8T_{1} \ge T_{0}^{2} + 4T_{5}^{2} + 4T_{0} T_{5} \Rightarrow 8T_{1} - T_{0}^{2} - 4T_{5}^{2} \ge 4T_{0} T_{5}$ or if $8\left( {\mu_{{p^{a} }}^{2} \left( {x_{i} } \right) + \mu_{{Q^{a} }}^{2} \left( {x_{i} } \right)} \right) - \mu_{{p^{a} }}^{2} \left( {x_{i} } \right) - \mu_{{Q^{a} }}^{2} \left( {x_{i} } \right) - 2\mu_{{P^{a} }} \left( {x_{i} } \right)\mu_{{Q^{a} }} \left( {x_{i} } \right) - 4\mu_{{P^{a} }} \left( {x_{i} } \right)\mu_{{Q^{a} }} \left( {x_{i} } \right) \ge 4\left( {\mu_{{P^{a} }} \left( {x_{i} } \right) + \mu_{{Q^{a} }} \left( {x_{i} } \right)} \right)\sqrt {\mu_{{P^{a} }} \left( {x_{i} } \right)\mu_{{Q^{a} }} \left( {x_{i} } \right)}$ or if $7\mu_{{p^{a} }}^{2} \left( {x_{i} } \right) + 7\mu_{{Q^{a} }}^{2} \left( {x_{i} } \right) - 6\mu_{{P^{a} }} \left( {x_{i} } \right)\mu_{{Q^{a} }} \left( {x_{i} } \right) \ge 4\left( {\mu_{{P^{a} }} \left( {x_{i} } \right) + \mu_{{Q^{a} }} \left( {x_{i} } \right)} \right)\sqrt {\mu_{{P^{a} }} \left( {x_{i} } \right)} \sqrt {\mu_{{Q^{a} }} \left( {x_{i} } \right)}$ or if $5\left( {\mu_{{p^{a} }}^{2} \left( {x_{i} } \right) + \mu_{{Q^{a} }}^{2} \left( {x_{i} } \right) - 2\mu_{{P^{a} }} \left( {x_{i} } \right)\mu_{{Q^{a} }} \left( {x_{i} } \right)} \right) + 2\mu_{{p^{a} }}^{2} \left( {x_{i} } \right) + 2\mu_{{Q^{a} }}^{2} \left( {x_{i} } \right) + 4\mu_{{P^{a} }} \left( {x_{i} } \right)\mu_{{Q^{a} }} \left( {x_{i} } \right) \ge 4\left( {\mu_{{P^{a} }} \left( {x_{i} } \right) + \mu_{{Q^{a} }} \left( {x_{i} } \right)} \right)\sqrt {\mu_{{P^{a} }} \left( {x_{i} } \right)} \sqrt {\mu_{{Q^{a} }} \left( {x_{i} } \right)}$ or if $5\left( {\mu_{{P^{a} }} \left( {x_{i} } \right) - \mu_{{Q^{a} }} \left( {x_{i} } \right)} \right)^{2} + 2\left( {\mu_{{P^{a} }} \left( {x_{i} } \right) + \mu_{{Q^{a} }} \left( {x_{i} } \right)} \right)^{2} \ge 4\left( {\mu_{{P^{a} }} \left( {x_{i} } \right) + \mu_{{Q^{a} }} \left( {x_{i} } \right)} \right)\sqrt {\mu_{{P^{a} }} \left( {x_{i} } \right)} \sqrt {\mu_{{Q^{a} }} \left( {x_{i} } \right)}$ or if $5\left( {\mu_{{P^{a} }} \left( {x_{i} } \right) - \mu_{{Q^{a} }} \left( {x_{i} } \right)} \right)^{2} + 2\left( {\mu_{{P^{a} }} \left( {x_{i} } \right) + \mu_{{Q^{a} }} \left( {x_{i} } \right)} \right)\left( {\mu_{{P^{a} }} \left( {x_{i} } \right) + \mu_{{Q^{a} }} \left( {x_{i} } \right) - 2\sqrt {\mu_{{P^{a} }} \left( {x_{i} } \right)} \sqrt {\mu_{{Q^{a} }} \left( {x_{i} } \right)} } \right) \ge 0$ or if $5\left( {\mu_{{P^{a} }} \left( {x_{i} } \right) - \mu_{{Q^{a} }} \left( {x_{i} } \right)} \right)^{2} + 2\left( {\mu_{{P^{a} }} \left( {x_{i} } \right) + \mu_{{Q^{a} }} \left( {x_{i} } \right)} \right)\left( {\sqrt {\mu_{{P^{a} }} \left( {x_{i} } \right)} - \sqrt {\mu_{{Q^{a} }} \left( {x_{i} } \right)} } \right)^{2} \ge 0$ which is true for each $\mu_{{P^{a} }} \left( {x_{i} } \right),\mu_{{Q^{a} }} \left( {x_{i} } \right) \in \left[ {0,1} \right]$ as desired.

Moreover, there exists the equality if $\mu_{{P^{a} }} \left( {x_{i} } \right) = \mu_{{Q^{a} }} \left( {x_{i} } \right)\forall i = 1,2,...,n.$

Thus, in view of Lemma 2.1, the resulting inequality $\sqrt {\frac{{T_{1} }}{2}} \ge \frac{{T_{2}^{2} }}{4}$ can be re-designed as $\frac{{T_{1} }}{2} \ge \frac{{T_{2}^{4} }}{16} \Rightarrow \frac{{T_{1} }}{2} + 1 \ge \frac{{T_{2}^{4} }}{16} + 1$

2b $$\Rightarrow \,\,\frac{{1 + T_{1} }}{{8 + T_{2}^{4} }} \ge \frac{1}{8}$$

Knowing the fact that sine hyperbolic function exhibits monotonicity over in [0,1], this implies (2b) to yield

3 $$\left( {2 + T_{0} } \right)\sinh \left( {\frac{{1 + T_{1} }}{{8 + T_{2}^{4} }}} \right) \ge \left( {2 + T_{0} } \right)\sinh \left( \frac{1}{8} \right)$$

On replacement of $\mu_{{P^{a} }} \left( {x_{i} } \right),\mu_{{Q^{a} }} \left( {x_{i} } \right)$ with their counterparts $1 - \mu_{{P^{a} }} \left( {x_{i} } \right),1 - \mu_{{Q^{a} }} \left( {x_{i} } \right)$,

$$T_{0} = \mu_{{P^{a} }} \left( {x_{i} } \right) + \mu_{{Q^{a} }} \left( {x_{i} } \right)$$ changes to $$1 - \mu_{{P^{a} }} \left( {x_{i} } \right) + 1 - \mu_{{Q^{a} }} \left( {x_{i} } \right) = 2 - \mu_{{P^{a} }} \left( {x_{i} } \right) - \mu_{{Q^{a} }} \left( {x_{i} } \right) = 2 - T_{0} ;$$

$$T_{1} = \mu_{{p^{a} }}^{2} \left( {x_{i} } \right) + \mu_{{Q^{a} }}^{2} \left( {x_{i} } \right) \to \left( {1 - \mu_{{P^{a} }} \left( {x_{i} } \right)} \right)^{2} + \left( {1 - \mu_{{Q^{a} }} \left( {x_{i} } \right)} \right)^{2} = T_{3} ;$$

$$T_{2} = \sqrt {\mu_{{P^{a} }} \left( {x_{i} } \right)} + \sqrt {\mu_{{Q^{a} }} \left( {x_{i} } \right)} \to \sqrt {1 - \mu_{{P^{a} }} \left( {x_{i} } \right)} + \sqrt {1 - \mu_{{Q^{a} }} \left( {x_{i} } \right)} = T_{4}$$

With these manipulations, the resulting inequality (3) reduces to

4 $$\left( {4 - T_{0} } \right)\sinh \left( {\frac{{1 + T_{3} }}{{8 + T_{4}^{4} }}} \right) \ge \left( {4 - T_{0} } \right)\sinh \left( \frac{1}{8} \right)$$

**Figure 2:**
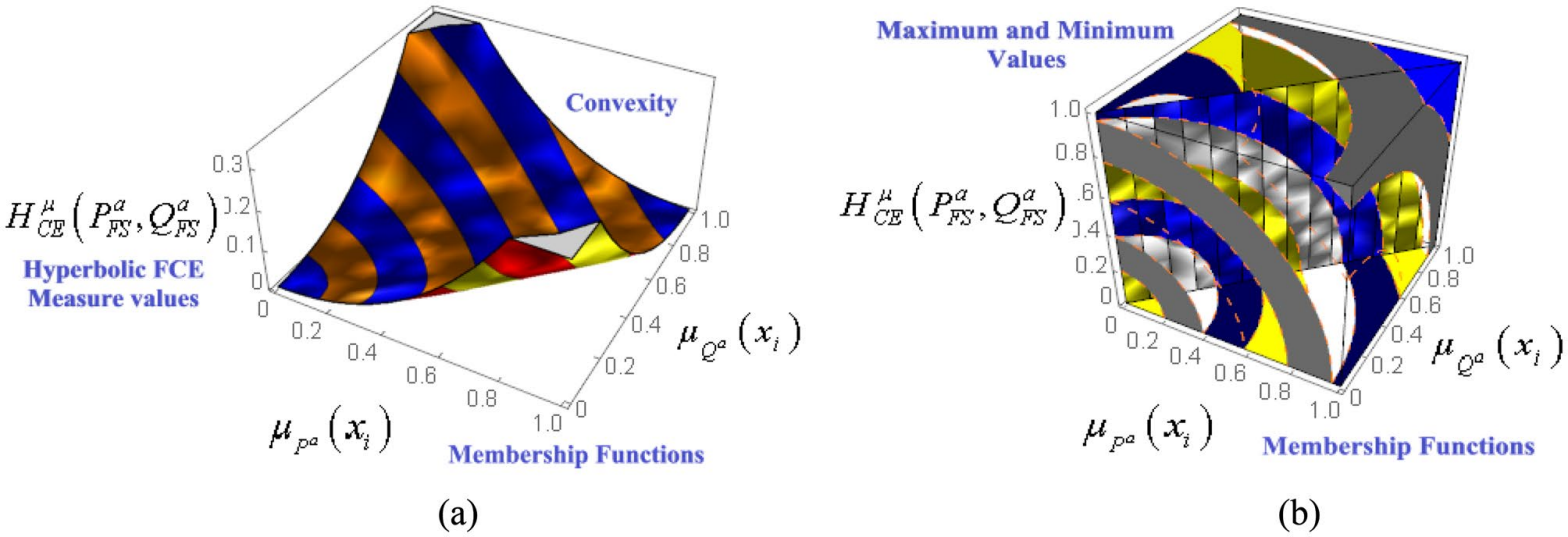
(**a**) Convexity property as indicated by $H_{CE}^{\mu } \left( {P_{FS}^{a} ,Q_{FS}^{a} } \right)$ (**b**) Extreme (Maximum and minimum) values as attained by $H_{CE}^{\mu } \left( {P_{FS}^{a} ,Q_{FS}^{a} } \right)$.

The desired result , that is, $\,H_{CE}^{\mu } \left( {P_{FS}^{a} ,Q_{FS}^{a} } \right) \ge 0\forall \mu_{{P^{a} }} \left( {x_{i} } \right),\mu_{{Q^{a} }} \left( {x_{i} } \right) \in \left[ {0,1} \right]$ can be easily obtained if we simply add the pro-offered inequalities (3, 4) and then take the summation over $i = 1$ to $i = n.$ Also, $H_{CE}^{\mu } \left( {P_{FS}^{a} ,Q_{FS}^{a} } \right)$ becomes zero when $\mu_{{P^{a} }} \left( {x_{i} } \right) = \mu_{{Q^{a} }} \left( {x_{i} } \right)\forall i = 1,2,...,n.$ The valid fact that our hyperbolic FCE measure meets the requirement of convexity property with respect to $\mu_{{P^{a} }} \left( {x_{i} } \right)$ and $\mu_{{Q^{a} }} \left( {x_{i} } \right)$ can be ensured from Fig. 2a.

We are now in a position to discuss the circumstances under which our proclaimed measure $H_{CE}^{\mu } \left( {P_{FS}^{a} ,Q_{FS}^{a} } \right)$ admits its extreme (maximum or minimum) value as justified in the following Theorem 2.3.

##### Theorem 2.3.

There exists the inequality: $0 \le \,H_{CE}^{\mu } \left( {P_{FS}^{a} ,Q_{FS}^{a} } \right) \le 6\left( {\sinh \frac{2}{9} - \sinh \frac{1}{8}} \right)n,$ where $n$ denotes the cardinality of $U = \left( {x_{1} ,x_{2} ,...,x_{n} } \right).$

##### Proof.

If we can replace $Q_{FS}^{a}$ with $C\left( {P_{FS}^{a} } \right)$ into (1), then

5 $$H_{CE}^{\mu } \left( {P_{FS}^{a} ,C\left( {P_{FS}^{a} } \right)} \right)\, = \sum\limits_{i = 1}^{n} {\left[ { - 6\sinh \left( \frac{1}{8} \right) + 6\sinh \left( {\frac{{1 + \mu_{{p^{a} }}^{2} \left( {x_{i} } \right) + \left( {1 - \mu_{{P^{a} }} \left( {x_{i} } \right)} \right)^{2} }}{{8 + \left( {\sqrt {\mu_{{P^{a} }} \left( {x_{i} } \right)} + \sqrt {1 - \mu_{{P^{a} }} \left( {x_{i} } \right)} } \right)^{4} }}} \right)\,} \right]}= \sum\limits_{i = 1}^{n} {\left[ {6\left( {\sinh \frac{2}{9} - \sinh \frac{1}{8}} \right) - 6\left( {\sinh \frac{2}{9} - \sinh \left( {\frac{{1 + \mu_{{p^{a} }}^{2} \left( {x_{i} } \right) + \left( {1 - \mu_{{P^{a} }} \left( {x_{i} } \right)} \right)^{2} }}{{8 + \left( {\sqrt {\mu_{{P^{a} }} \left( {x_{i} } \right)} + \sqrt {1 - \mu_{{P^{a} }} \left( {x_{i} } \right)} } \right)^{4} }}} \right)} \right)\,} \right]} \, = 6\,Max.H_{FS} \left( {P_{FS}^{a} } \right) - 6H_{FS} \left( {P_{FS}^{a} } \right)$$

In view of resulting Theorem 2.1, $H_{FS} \left( {P_{FS}^{a} } \right) \ge 0\forall P_{FS}^{a} \in F\left( U \right)$ and hence (5) yields

6 $$0 \le H_{CE}^{\mu } \left( {P_{FS}^{a} ,C\left( {P_{FS}^{a} } \right)} \right)\, \le 6\left( {\sinh \frac{2}{9} - \sinh \frac{1}{8}} \right)n$$

Because $n$ is a natural number, (6) clarifies that $H_{CE}^{\mu } \left( {P_{FS}^{a} ,C\left( {P_{FS}^{a} } \right)} \right)\,$ is a finite entity which is bounded by two real numbers. Consecutively our proclaimed entropy measure $H_{CE}^{\mu } \left( {P_{FS}^{a} ,Q_{FS}^{a} } \right)$ is also a finite entity and bounded by two real numbers, which are respectively the min/max values of $H_{CE}^{\mu } \left( {P_{FS}^{a} ,Q_{FS}^{a} } \right).$ Equation (6) indicates that the maximum value is independent of the entities of $U$, but depends on $n$. The convexity property indicated by $H_{CE}^{\mu } \left( {P_{FS}^{a} ,Q_{FS}^{a} } \right)$ ensures the fact that our proposed hyperbolic FCE measure exhibits its minimum value, which is zero. Also, Fig. 2b makes it clear that our $H_{CE}^{\mu } \left( {P_{FS}^{a} ,Q_{FS}^{a} } \right)$ should increase whenever the absolute difference $\left| {P_{FS}^{a} - Q_{FS}^{a} \,} \right|$ reaches its maximum: $6\left( {\sinh \frac{2}{9} - \sinh \frac{1}{8}} \right)n$ at $\left( {1,0} \right)$ &$\left( {0,1} \right)$ and minimum at $\left( {0,0} \right).$

To identify the possible relationship between winding faults and frequency ranges, it is necessary to classify mechanical faults (AD and RD) as well as SC faults. The desired goal can be achieved by deploying the proclaimed hyperbolic fuzzy cross entropy measure as follows.

## Fuzzy cross entropy based distinction and classification of transformer winding defects procedure

In order to introduce how the suggested technique is used to resolve the fault diagnosis issue, it is defined in multiple steps.

*Step 1* OMICRON FRANO 800 SFRA Analyzer is used to measure frequency response

Different experiments are applied to a transformer, and an omicron FRANEO 800 analyser is utilized to measure its FRA under reference data (healthy) and different fault conditions. Moreover, the FRA's measured spectra is classified into three main sub-bands including high-, middle-, and low-frequency band that are > 600, 100–600, and < 100 kHz, respectively.

*Step 2* Frequency response normalization

Before fuzzification, it becomes necessary to represent a proper clustering method and improve the accuracy of FRA to normalize the obtained frequency responses. m (= 3) and n (= 30) present the number of frequency bands and the number of fault levels, respectively. Moreover, *v*_*ji*_ denotes the monitored frequency responses of ith frequency band at the *j*th fault level. Frequency responses normalization of healthy and fault conditions in [0,1] interval is mandatory before fuzzification. If $V_{ji}$ is normalized frequency responses, then

7 $$V_{ji} = \frac{{v_{ji} - {\text{Min}}.v_{ji} }}{{{\text{Max}}.v_{ji} - {\text{Min}}.v_{ji} }};j = 1,2,...n\left( { = 30} \right),i = 1,2,...,m\left( { = 3} \right).$$

*Step 3* Lower band extraction

In this study, 30 fault levels are simulated that the first, second, and third ten fault levels present the SC, AD, and RD faults, respectively. After normalizing the frequency responses in different fault, it is time to extract the lower bounds in each frequency band from the normalized frequency responses. The lower bounds are considered as degrees of truth membership. Let $\tilde{\mu }_{j} \left( {x_{i} } \right)$ show the truth membership degree extracted from the normalized FRAs of *i*th frequency band at the *j*th fault level. Then

8 $$\tilde{\mu }_{j} \left( {x_{i} } \right) = {\text{Min}}{.}V_{ji} \left( {j = 1,2,3,...,30;i = 1,2,3} \right)$$

This work calculates the truth membership degrees for SC, AD, RD faults by Eq. (7).

*Step 4* Fuzzy Set Construction

For experiencing macroscopic categorization of fault conditions of the winding, the resulting lower bounds should be converted into fuzzy sets. This conversion is systematic and can be done as follows. Different transformer winding fault conditions are represented by $A_{K} \left( {K = 1,2,3} \right)$ where *A*_*1*_ , *A*_*2*_, and *A*_*3*_ show the SC, AD, RD faults, respectively. These values are denoted by Eqs. (9, (10), 11). Thus

9 $$A_{\,1} = \left( { < x_{i} ,\tilde{\mu }_{j} \left( {x_{i} } \right) > } \right), \;\;j = 1,2,...,10;\;\;i = 1,2,3.$$

10 $$A_{\,2} = \left( { < x_{i} ,\tilde{\mu }_{j} \left( {x_{i} } \right) > } \right),\;\;j = 11,12,...,20\,\;\;i = 1,2,3.$$

11 $$A_{\,3} = \left( { < x_{i} ,\tilde{\mu }_{j} \left( {x_{i} } \right) > } \right),\;\;j = 21,22,...,30;\;\;i = 1,2,3.$$

*Step 5* Calculating measure values of hyperbolic fuzzy cross entropy

Moreover, the Eq. (1) can be used to calculate the hyperbolic FCE measure values between the predefined fuzzy sets $A_{1} ,B_{1} ;A_{2} ,B_{2} ;A_{3} ,B_{3}$ as bellows. Therefore, the suggested hyperbolic fuzzy cross entropy measure between different fault status (SC, AD and RD) and healthy state can be represented by the expressions $H_{CE}^{\mu } \left( {A_{1} ,B_{1} } \right),\,H_{CE}^{\mu } \left( {A_{2} ,B_{2} } \right),\,H_{CE}^{\mu } \left( {A_{3} ,B_{3} } \right)$ respectively which can be gained by substitution $\mu_{{P^{a} }} \left( {x_{i} } \right)$ with $\tilde{\mu }_{{A_{K} }} \left( {x_{i} } \right)\left( {K = 1,2,3} \right)$ and $\mu_{{Q^{a} }} \left( {x_{i} } \right)$ with $\tilde{\mu }_{{B_{K} }} \left( {x_{i} } \right)\left( {K = 1,2,3} \right)$ into (1). Thus

12 $$H_{CE}^{\mu } \left( {A_{1} ,B_{1} } \right) = \sum\limits_{i = 1}^{10} {\left[ \begin{gathered} - 6\sinh \left( \frac{1}{8} \right) + \left( {2 + \tilde{\mu }_{{A_{1} }} \left( {x_{i} } \right) + \tilde{\mu }_{{B_{1} }} \left( {x_{i} } \right)} \right)\sinh \,\left( {\frac{{1 + \tilde{\mu }_{{A_{1} }}^{2} \left( {x_{i} } \right) + \tilde{\mu }_{{B_{1} }}^{2} \left( {x_{i} } \right)\left( {x_{i} } \right)}}{{8 + \left( {\sqrt {\tilde{\mu }_{{A_{1} }} \left( {x_{i} } \right)} + \sqrt {\tilde{\mu }_{{B_{1} }} \left( {x_{i} } \right)} } \right)^{4} }}} \right)\, \hfill \\ + \left( {4 - \tilde{\mu }_{{A_{1} }} \left( {x_{i} } \right) - \tilde{\mu }_{{B_{1} }} \left( {x_{i} } \right)} \right)\sinh \,\left( {\frac{{1 + \left( {1 - \tilde{\mu }_{{A_{1} }} \left( {x_{i} } \right)} \right)^{2} + \left( {1 - \tilde{\mu }_{{B_{1} }} \left( {x_{i} } \right)} \right)^{2} }}{{8 + \left( {\tilde{\mu }_{{A_{1} }} \left( {x_{i} } \right) + \sqrt {1 - \tilde{\mu }_{{B_{1} }} \left( {x_{i} } \right)} } \right)^{4} }}} \right) \hfill \\ \end{gathered} \right]} \,\,$$

13 $$H_{CE}^{\mu } \left( {A_{2} ,B_{2} } \right) = \sum\limits_{i = 11}^{20} {\left[ \begin{gathered} - 6\sinh \left( \frac{1}{8} \right) + \left( {2 + \tilde{\mu }_{{A_{2} }} \left( {x_{i} } \right) + \tilde{\mu }_{{B_{2} }} \left( {x_{i} } \right)} \right)\sinh \,\left( {\frac{{1 + \tilde{\mu }_{{A_{2} }}^{2} \left( {x_{i} } \right) + \tilde{\mu }_{{B_{2} }}^{2} \left( {x_{i} } \right)\left( {x_{i} } \right)}}{{8 + \left( {\sqrt {\tilde{\mu }_{{A_{2} }} \left( {x_{i} } \right)} + \sqrt {\tilde{\mu }_{{B_{2} }} \left( {x_{i} } \right)} } \right)^{4} }}} \right)\, \hfill \\ + \left( {4 - \tilde{\mu }_{{A_{2} }} \left( {x_{i} } \right) - \tilde{\mu }_{{B_{2} }} \left( {x_{i} } \right)} \right)\sinh \,\left( {\frac{{1 + \left( {1 - \tilde{\mu }_{{A_{2} }} \left( {x_{i} } \right)} \right)^{2} + \left( {1 - \tilde{\mu }_{{B_{2} }} \left( {x_{i} } \right)} \right)^{2} }}{{8 + \left( {\tilde{\mu }_{{A_{2} }} \left( {x_{i} } \right) + \sqrt {1 - \tilde{\mu }_{{B_{2} }} \left( {x_{i} } \right)} } \right)^{4} }}} \right) \hfill \\ \end{gathered} \right]} \,\,$$

14 $$H_{CE}^{\mu } \left( {A_{3} ,B_{3} } \right) = \sum\limits_{i = 21}^{30} {\left[ \begin{gathered} - 6\sinh \left( \frac{1}{8} \right) + \left( {2 + \tilde{\mu }_{{A_{3} }} \left( {x_{i} } \right) + \tilde{\mu }_{{B_{3} }} \left( {x_{i} } \right)} \right)\sinh \,\left( {\frac{{1 + \tilde{\mu }_{{A_{3} }}^{2} \left( {x_{i} } \right) + \tilde{\mu }_{{B_{3} }}^{2} \left( {x_{i} } \right)\left( {x_{i} } \right)}}{{8 + \left( {\sqrt {\tilde{\mu }_{{A_{3} }} \left( {x_{i} } \right)} + \sqrt {\tilde{\mu }_{{B_{3} }} \left( {x_{i} } \right)} } \right)^{4} }}} \right)\, \hfill \\ + \left( {4 - \tilde{\mu }_{{A_{3} }} \left( {x_{i} } \right) - \tilde{\mu }_{{B_{3} }} \left( {x_{i} } \right)} \right)\sinh \,\left( {\frac{{1 + \left( {1 - \tilde{\mu }_{{A_{3} }} \left( {x_{i} } \right)} \right)^{2} + \left( {1 - \tilde{\mu }_{{B_{3} }} \left( {x_{i} } \right)} \right)^{2} }}{{8 + \left( {\tilde{\mu }_{{A_{3} }} \left( {x_{i} } \right) + \sqrt {1 - \tilde{\mu }_{{B_{3} }} \left( {x_{i} } \right)} } \right)^{4} }}} \right) \hfill \\ \end{gathered} \right]} \,\,$$

The Bhandari and Pal^23^ fuzzy cross entropy measure between different fault (SC, AD and RD) and healthy condition can be similarly expressed as $H_{B}^{\mu } \left( {A_{1} ,B_{1} } \right),\,H_{B}^{\mu } \left( {A_{2} ,B_{2} } \right),\,H_{B}^{\mu } \left( {A_{3} ,B_{3} } \right)$ respectively and can be similarly obtained as:

15 $$H_{B}^{\mu } \left( {A_{1} ,B_{1} } \right) = \sum\limits_{j = 1}^{10} {\left[ {\tilde{\mu }_{{A_{1} }} \left( {x_{i} } \right)\log \left( {\frac{{\tilde{\mu }_{{A_{1} }} \left( {x_{i} } \right)}}{{\tilde{\mu }_{{B_{1} }} \left( {x_{i} } \right)}}} \right) + \left( {1 - \tilde{\mu }_{{A_{1} }} \left( {x_{i} } \right)} \right)\log \left( {\frac{{1 - \tilde{\mu }_{{A_{1} }} \left( {x_{i} } \right)}}{{1 - \tilde{\mu }_{{B_{1} }} \left( {x_{i} } \right)}}} \right)} \right]} \,.$$

16 $$H_{B}^{\mu } \left( {A_{2} ,B_{2} } \right) = \sum\limits_{j = 11}^{20} {\left[ {\tilde{\mu }_{{A_{2} }} \left( {x_{i} } \right)\log \left( {\frac{{\tilde{\mu }_{{A_{2} }} \left( {x_{i} } \right)}}{{\tilde{\mu }_{{B_{2} }} \left( {x_{i} } \right)}}} \right) + \left( {1 - \tilde{\mu }_{{A_{2} }} \left( {x_{i} } \right)} \right)\log \left( {\frac{{1 - \tilde{\mu }_{{A_{2} }} \left( {x_{i} } \right)}}{{1 - \tilde{\mu }_{{B_{2} }} \left( {x_{i} } \right)}}} \right)} \right]} \,.$$

17 $$H_{B}^{\mu } \left( {A_{3} ,B_{3} } \right) = \sum\limits_{j = 21}^{30} {\left[ {\tilde{\mu }_{{A_{3} }} \left( {x_{i} } \right)\log \left( {\frac{{\tilde{\mu }_{{A_{3} }} \left( {x_{i} } \right)}}{{\tilde{\mu }_{{B_{3} }} \left( {x_{i} } \right)}}} \right) + \left( {1 - \tilde{\mu }_{{A_{3} }} \left( {x_{i} } \right)} \right)\log \left( {\frac{{1 - \tilde{\mu }_{{A_{3} }} \left( {x_{i} } \right)}}{{1 - \tilde{\mu }_{{B_{3} }} \left( {x_{i} } \right)}}} \right)} \right]} \,.$$

Similarly, the Shang and Jiang^23^ fuzzy cross entropy between different faults and healthy conditions can be expressed $H_{S}^{\mu } \left( {A_{1} ,B_{1} } \right),\,H_{S}^{\mu } \left( {A_{2} ,B_{2} } \right),\,H_{S}^{\mu } \left( {A_{3} ,B_{3} } \right)$ respectively and can be obtained as:

18 $$H_{S}^{\mu } \left( {A_{1} ,B_{1} } \right) = \sum\limits_{j = 1}^{10} {\left[ {\tilde{\mu }_{{A_{1} }} \left( {x_{i} } \right)\log \left( {\frac{{2\tilde{\mu }_{{A_{1} }} \left( {x_{i} } \right)}}{{\tilde{\mu }_{{A_{1} }} \left( {x_{i} } \right) + \tilde{\mu }_{{B_{1} }} \left( {x_{i} } \right)}}} \right) + \left( {1 - \tilde{\mu }_{{A_{1} }} \left( {x_{i} } \right)} \right)\log \left( {\frac{{1 - \tilde{\mu }_{{A_{1} }} \left( {x_{i} } \right)}}{{1 - \frac{1}{2}\left( {\tilde{\mu }_{{A_{1} }} \left( {x_{i} } \right) + \tilde{\mu }_{{B_{1} }} \left( {x_{i} } \right)} \right)}}} \right)} \right]} \,.$$

19 $$H_{S}^{\mu } \left( {A_{2} ,B_{2} } \right) = \sum\limits_{j = 11}^{20} {\left[ {\tilde{\mu }_{{A_{2} }} \left( {x_{i} } \right)\log \left( {\frac{{2\tilde{\mu }_{{A_{2} }} \left( {x_{i} } \right)}}{{\tilde{\mu }_{{A_{2} }} \left( {x_{i} } \right) + \tilde{\mu }_{{B_{2} }} \left( {x_{i} } \right)}}} \right) + \left( {1 - \tilde{\mu }_{{A_{1} }} \left( {x_{i} } \right)} \right)\log \left( {\frac{{1 - \tilde{\mu }_{{A_{2} }} \left( {x_{i} } \right)}}{{1 - \frac{1}{2}\left( {\tilde{\mu }_{{A_{2} }} \left( {x_{i} } \right) + \tilde{\mu }_{{B_{2} }} \left( {x_{i} } \right)} \right)}}} \right)} \right]} \,.$$

20 $$H_{S}^{\mu } \left( {A_{3} ,B_{3} } \right) = \sum\limits_{j = 21}^{30} {\left[ {\tilde{\mu }_{{A_{3} }} \left( {x_{i} } \right)\log \left( {\frac{{2\tilde{\mu }_{{A_{3} }} \left( {x_{i} } \right)}}{{\tilde{\mu }_{{A_{3} }} \left( {x_{i} } \right)\tilde{\mu }_{{B_{3} }} \left( {x_{i} } \right)}}} \right) + \left( {1 - \tilde{\mu }_{{A_{3} }} \left( {x_{i} } \right)} \right)\log \left( {\frac{{1 - \tilde{\mu }_{{A_{3} }} \left( {x_{i} } \right)}}{{1 - \frac{1}{2}\left( {\tilde{\mu }_{{A_{3} }} \left( {x_{i} } \right) + \tilde{\mu }_{{B_{3} }} \left( {x_{i} } \right)} \right)}}} \right)} \right]} \,.$$

*Step 6* Fault condition identification

**Figure 3:**
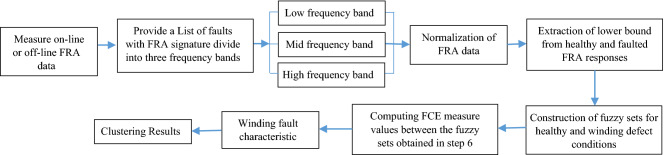
The suggested procedure for the distinction and taxonomy of faults.

The highest FCE measure value between the fuzzy sets of faulted and healthy conditions confirms the fault occurrence in winding in a proper frequency band. Figure 3 demonstrates the suggested method’s algorithm for transformer winding fault classification and discrimination.

## Case study

### Power transformer data set

This study employs a 20/0.4 kV, 1200KVA, three-phase transformer. The Low- and High-Voltage windings are made up of an interleaved disks and a continuous layer, respectively. Mineral oil and Kraft paper form the insulation of the transformer. In this arrangement, internal nodes should be accessed. Therefore, on each winding, the measurement of each fault is separately done. In this study, in each of the phases (A, B and C), ten levels of each defect (including SC, RD and AD) were simulated artificially in different places and levels, respectively.

**Figure 4:**
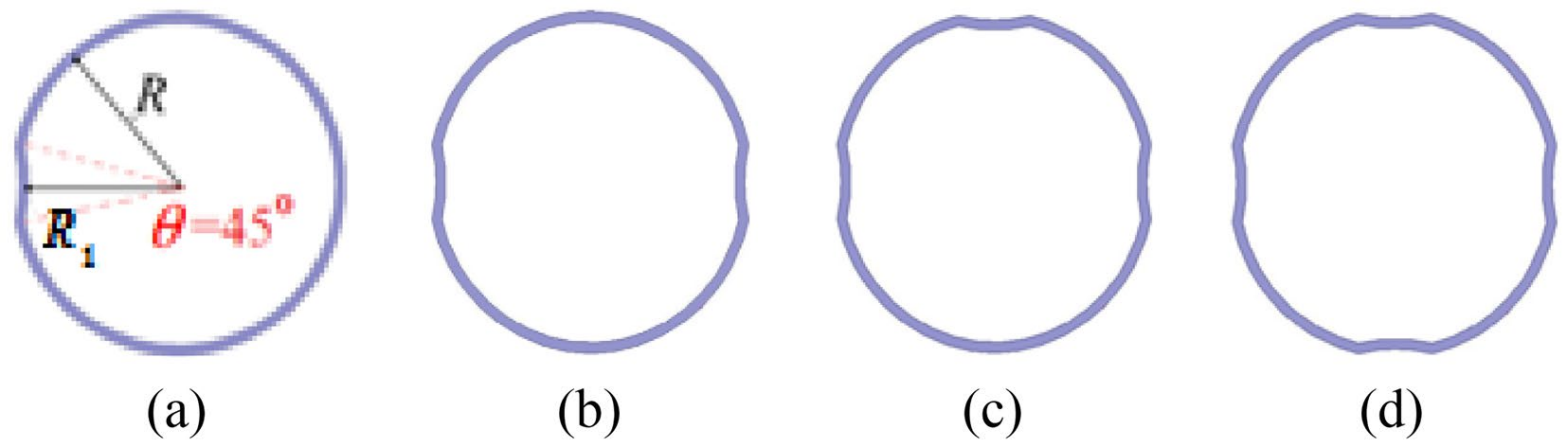
(**a**) one- (**b**) two- (**c**) three- (**d**) four-directions of winding radial deformation.

- *Short Circuit:* A part of the high voltage windings of the transformer have been short-circuited to implement different levels of short circuit in different locations. The SC faults are simulated as Table 1:
- *Axial Displacement (AD):* Here, to simulate this displacement at ten different levels, the high voltage winding is displaced 64 mm (in different steps according Table 2) relative to the low voltage winding to specify the effect of this fault on the FRA. These faults are simulated as Table 2:
- *Radial Deformation:* To simulate this fault, we apply deformations in ten levels on the disc winding and Table 3. Figure 4 illustrated a view of radial deformations of the winding in different directions. R_1_, R, and d (d = R-R1) represent respectively the minimum average radius and the average radius, and the radial deformation amount, which is variable. The angle is denoted by *Θ* that at 45° is fixed. Moreover, to apply various levels of RD simulated in various direction, the ratio $\frac{d}{R}$ is set to 2, 4, and 7% (Fig. 4a–d). The RD fault level percentage is calculated in Table 3:
  21 $$\% {\text{RD}}\;{\text{Fault}}\;{\text{Level}}\; = \frac{{R - R_{1} }}{R} \times 100\% = \frac{d}{R} \times 100\%$$

**Table 1:** Different short circuit levels at various locations.

Fault No	The location of connector
SC_1_	Between disk 11 and disk 12
SC_2_	Between disk 18 and disk 20
SC_3_	Between disk 11–12 and 25–26
SC_4_	Between disk 25 and disk 26
SC_5_	Between disk 32 and disk 35
SC_6_	Between disk 52 and disk 54
SC_7_	Between disk 58 and disk 60
SC_8_	Between disk 65 and disk 68
SC_9_	Between disk 58–60 and 52–54
SC_10_	Between disk 48 and disk 50

**Table 2:** Various levels of the axial displacement.

Fault No	The displacement in mm
AD_1_	7
AD_2_	12
AD_3_	19
AD_4_	24
AD_5_	31
AD_6_	39
AD_7_	45
AD_8_	50
AD_9_	57
AD_10_	64

**Table 3:** Various levels of the radial deformation.

Fault No	Percentage of Deformation	Location
RD_1_	2% (1 side)	The 11th up to the 35th disks
RD_2_	4% (1 side)	
RD_3_	7% (1 side)	
RD_4_	2% (2 opposite sides)	
RD_5_	4% (2 opposite sides)	
RD_6_	7% (2 opposite sides)	
RD_7_	2% (3 sides)	
RD_8_	4% (3 sides)	
RD_9_	2% (4 sides)	
RD_10_	4% (4 sides)

Table 4 shows the 1.2 MVA power transformer specifications and dimensions.

**Table 4:** Specifications of the transformer under study.

Parameter	Value
Impedance (%), Frequency (Hz), Phase	2.431, 50, 3
Rated power (KVA)	1200
Primary voltage /Secondary voltage (KV)	20/0.4
Low voltage/High voltage winding Turns	112/5600
Outer/Inner HV winding diameter (mm)	1086/987
Outer/Inner LV winding diameter (mm)	891/823
Height of primary/Secondary windings (mm)	1154/1249
Core height /Length (mm)	2033/3785
Disk number of HV winding	70

### FRA simulation results

**Figure 5:**
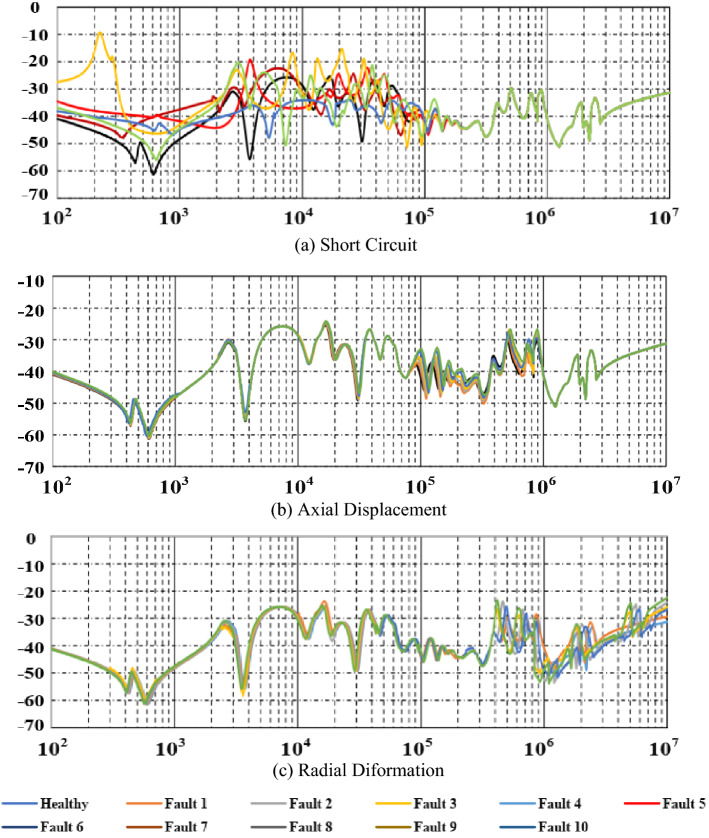
The effect of RD, AD, and SC defects in FRA for different levels of faults.

Figure 5a–c illustrates the effect of RD, AD, and SC defects on the voltage waveform of the transformer in ten different fault levels. In this study, the measurement of the FRA performs by an OMICRON analyser. As seen in Fig. 5, despite the FRA trace variance, it is very difficult to analyse. Furthermore, conventional FRA has difficulty recognizing low levels of fault. However, our proposed methodology to automate the interpretation procedure can be easily performed in FRA as follows.

### Distinction and identification of transformer winding faults

We shall now employ our suggested fuzzy cross entropy measure-based method for fault detection and classification. This technique increases the ability to visually diagnose defect and classify various winding faults and enhance the interpretation accuracy of FRA signatures. Moreover, for interpreting and taxonomy the frequency response results, the FRA spectrum is categorized into three main sub-bands, including high-, middle-, and low-frequency bands that are > 600, 100–600, and < 100 kHz, respectively. In this research, 30 levels of fault are simulated that the first, second, and third ten fault levels present the SC, AD, and RD faults, respectively, which these faults can be represented by the set $A_{1} = \left( {F_{1} ,F_{2} ,...,F_{10} } \right).$, $\,A_{2} = \left( {F_{11} ,F_{12} ,...,F_{20} } \right)$, $A_{3} = \left( {F_{21} ,F_{22} ,...,F_{30} } \right).$ We have extracted the truth membership degrees from the normalized frequency responses of short circuit, AD and RD faults types in the low, medium and high frequency ranges. The results are displayed in Table 5.

**Table 5:** Extraction of lower bounds for SC, AD and RD fault conditions in pre-defined frequency bands.

Low Frequency						Middle frequency						High frequency					
SC		AD		RD		SC		AD		RD		SC		AD		RD	
F1	0.5875	F11	0.3675	F21	0.3646	F1	0.2947	F11	0.4505	F21	0.3730	F1	0.3730	F11	0.3773	F21	0.5283
F2	0.5629	F12	0.3706	F22	0.3620	F2	0.2819	F12	0.4785	F22	0.3722	F2	0.3922	F12	0.4083	F22	0.5302
F3	0.5982	F13	0.3729	F23	0.3606	F3	0.2921	F13	0.4756	F23	0.3730	F3	0.3730	F13	0.4221	F23	0.5664
F4	0.5811	F14	0.3735	F24	0.3597	F4	0.2947	F14	0.4945	F24	0.3730	F4	0.3730	F14	0.3730	F24	0.5891
F5	0.5658	F15	0.3775	F25	0.3585	F5	0.3014	F15	0.4789	F25	0.3918	F5	0.3918	F15	0.3730	F25	0.5485
F6	0.5764	F16	0.3729	F26	0.3746	F6	0.2815	F16	0.4534	F26	0.3736	F6	0.4136	F16	0.4030	F26	0.5763
F7	0.5456	F17	0.3570	F27	0.3646	F7	0.3069	F17	0.4818	F27	0.3730	F7	0.4073	F17	0.3913	F27	0.5634
F8	0.5697	F18	0.3646	F28	0.3542	F8	0.3012	F18	0.4642	F28	0.3742	F8	0.3891	F18	0.3703	F28	0.5792
F9	0.5654	F19	0.3646	F29	0.3723	F9	0.3118	F19	0.4743	F29	0.3802	F9	0.3802	F19	0.3595	F29	0.5401
F10	0.5862	F20	0.3596	F30	0.3646	F10	0.3120	F20	0.4569	F30	0.3918	F10	0.4218	F20	0.4106	F30	0.5246

After extracting the lower bounds from the normalized FRAs of different types of faults in the predefined frequency bands, our next goal is to employ the resulting Eqs. (12), (13), (14) for computing hyperbolic fuzzy cross entropy measure $H_{CE}^{\mu } \left( {F_{i} ,\,0.3646} \right);i = 1,2,...,30$ values which arises between the fuzzy sets of fault types and healthy condition in the low, middle and high frequency bands consecutively. Here, the truth membership degrees for the healthy condition in low, medium and high frequency ranges have been computed as 0.3646, 0.2947, and 0.373, respectively. The results are displayed in Tables 6, 7 and 8. For instance, in *low-frequency* band, we have computed

**Table 6:** Computation of FCE measure values between the fuzzy sets of various fault types in low frequency range.

SC	$H_{CE}^{\mu } \left( {F_{1} ,\,0.3646} \right)$	0.01867	$H_{CE}^{\mu } \left( {F_{2} ,\,0.3646} \right)$	0.01474	$H_{CE}^{\mu } \left( {F_{3} ,\,0.3646} \right)$	0.02053	$H_{CE}^{\mu } \left( {F_{4} ,\,0.3646} \right)$	0.01760	$H_{CE}^{\mu } \left( {F_{5} ,\,0.3646} \right)$	0.01518
	$H_{CE}^{\mu } \left( {F_{6} ,\,0.3646} \right)$	0.01684	$H_{CE}^{\mu } \left( {F_{7} ,\,0.3646} \right)$	0.01226	$H_{CE}^{\mu } \left( {F_{8} ,\,0.3646} \right)$	0.01578	$H_{CE}^{\mu } \left( {F_{9} ,\,0.3646} \right)$	0.01512	$H_{CE}^{\mu } \left( {F_{10} ,\,0.3646} \right)$	0.01845
AD	$H_{CE}^{\mu } \left( {F_{11} ,\,0.3646} \right)$	0.00000	$H_{CE}^{\mu } \left( {F_{12} ,\,0.3646} \right)$	0.00001	$H_{CE}^{\mu } \left( {F_{13} ,\,0.3646} \right)$	0.00003	$H_{CE}^{\mu } \left( {F_{14} ,\,0.3646} \right)$	0.00003	$H_{CE}^{\mu } \left( {F_{15} ,\,0.3646} \right)$	0.00006
	$H_{CE}^{\mu } \left( {F_{16} ,\,0.3646} \right)$	0.00003	$H_{CE}^{\mu } \left( {F_{17} ,\,0.3646} \right)$	0.00002	$H_{CE}^{\mu } \left( {F_{18} ,\,0.3646} \right)$	0.00000	$H_{CE}^{\mu } \left( {F_{19} ,\,0.3646} \right)$	0.00000	$H_{CE}^{\mu } \left( {F_{20} ,\,0.3646} \right)$	0.00001
RD	$H_{CE}^{\mu } \left( {F_{21} ,\,0.3646} \right)$	0.00000	$H_{CE}^{\mu } \left( {F_{22} ,\,0.3646} \right)$	0.00000	$H_{CE}^{\mu } \left( {F_{23} ,\,0.3646} \right)$	0.00001	$H_{CE}^{\mu } \left( {F_{24} ,\,0.3646} \right)$	0.00001	$H_{CE}^{\mu } \left( {F_{25} ,\,0.3646} \right)$	0.00001
	$H_{CE}^{\mu } \left( {F_{26} ,\,0.3646} \right)$	0.00004	$H_{CE}^{\mu } \left( {F_{27} ,\,0.3646} \right)$	0.00000	$H_{CE}^{\mu } \left( {F_{28} ,\,0.3646} \right)$	0.00004	$H_{CE}^{\mu } \left( {F_{29} ,\,0.3646} \right)$	0.00002	$H_{CE}^{\mu } \left( {F_{30} ,\,0.3646} \right)$	0.00000

**Table 7:** Computation of FCE measure values between the fuzzy sets of various fault types in middle frequency range.

SC	$H_{CE}^{\mu } \left( {F_{1} ,\,0.2947} \right)$	0.00000	$H_{CE}^{\mu } \left( {F_{2} ,\,0.2947} \right)$	0.00006	$H_{CE}^{\mu } \left( {F_{3} ,\,0.2947} \right)$	0.00000	$H_{CE}^{\mu } \left( {F_{4} ,\,0.2947} \right)$	0.00000	$H_{CE}^{\mu } \left( {F_{5} ,\,0.2947} \right)$	0.00002
	$H_{CE}^{\mu } \left( {F_{6} ,\,0.2947} \right)$	0.00006	$H_{CE}^{\mu } \left( {F_{7} ,\,0.2947} \right)$	0.00005	$H_{CE}^{\mu } \left( {F_{8} ,\,0.2947} \right)$	0.00002	$H_{CE}^{\mu } \left( {F_{9} ,\,0.2947} \right)$	0.00011	$H_{CE}^{\mu } \left( {F_{10} ,\,0.2947} \right)$	0.00011
AD	$H_{CE}^{\mu } \left( {F_{11} ,\,0.2947} \right)$	0.00901	$H_{CE}^{\mu } \left( {F_{12} ,\,0.2947} \right)$	0.01259	$H_{CE}^{\mu } \left( {F_{13} ,\,0.2947} \right)$	0.01219	$H_{CE}^{\mu } \left( {F_{14} ,\,0.2947} \right)$	0.01491	$H_{CE}^{\mu } \left( {F_{15} ,\,0.2947} \right)$	0.01264
	$H_{CE}^{\mu } \left( {F_{16} ,\,0.2947} \right)$	0.00935	$H_{CE}^{\mu } \left( {F_{17} ,\,0.2947} \right)$	0.01305	$H_{CE}^{\mu } \left( {F_{18} ,\,0.2947} \right)$	0.01068	$H_{CE}^{\mu } \left( {F_{19} ,\,0.2947} \right)$	0.01201	$H_{CE}^{\mu } \left( {F_{20} ,\,0.2947} \right)$	0.00978
RD	$H_{CE}^{\mu } \left( {F_{21} ,\,0.2947} \right)$	0.00000	$H_{CE}^{\mu } \left( {F_{22} ,\,0.2947} \right)$	0.00000	$H_{CE}^{\mu } \left( {F_{23} ,\,0.2947} \right)$	0.00000	$H_{CE}^{\mu } \left( {F_{24} ,\,0.2947} \right)$	0.00000	$H_{CE}^{\mu } \left( {F_{25} ,\,0.2947} \right)$	0.00008
	$H_{CE}^{\mu } \left( {F_{26} ,\,0.2947} \right)$	0.00004	$H_{CE}^{\mu } \left( {F_{27} ,\,0.2947} \right)$	0.00007	$H_{CE}^{\mu } \left( {F_{28} ,\,0.2947} \right)$	0.00002	$H_{CE}^{\mu } \left( {F_{29} ,\,0.2947} \right)$	0.00012	$H_{CE}^{\mu } \left( {F_{30} ,\,0.2947} \right)$	0.00001

**Table 8:** Computation of FCE measure values between the fuzzy sets of various fault types in high frequency range.

SC	$H_{CE}^{\mu } \left( {F_{1} ,\,0.373} \right)$	0.00000	$H_{CE}^{\mu } \left( {F_{2} ,\,0.373} \right)$	0.00014	$H_{CE}^{\mu } \left( {F_{3} ,\,0.373} \right)$	0.00000	$H_{CE}^{\mu } \left( {F_{4} ,\,0.373} \right)$	0.00000	$H_{CE}^{\mu } \left( {F_{5} ,\,0.373} \right)$	0.00013
	$H_{CE}^{\mu } \left( {F_{6} ,\,0.373} \right)$	0.00061	$H_{CE}^{\mu } \left( {F_{7} ,\,0.373} \right)$	0.00044	$H_{CE}^{\mu } \left( {F_{8} ,\,0.373} \right)$	0.00010	$H_{CE}^{\mu } \left( {F_{9} ,\,0.373} \right)$	0.00002	$H_{CE}^{\mu } \left( {F_{10} ,\,0.373} \right)$	0.00088
AD	$H_{CE}^{\mu } \left( {F_{11} ,\,0.373} \right)$	0.00001	$H_{CE}^{\mu } \left( {F_{12} ,\,0.373} \right)$	0.00046	$H_{CE}^{\mu } \left( {F_{13} ,\,0.373} \right)$	0.00089	$H_{CE}^{\mu } \left( {F_{14} ,\,0.373} \right)$	0.00000	$H_{CE}^{\mu } \left( {F_{15} ,\,0.373} \right)$	0.00000
	$H_{CE}^{\mu } \left( {F_{16} ,\,0.373} \right)$	0.00033	$H_{CE}^{\mu } \left( {F_{17} ,\,0.373} \right)$	0.00012	$H_{CE}^{\mu } \left( {F_{18} ,\,0.373} \right)$	0.00000	$H_{CE}^{\mu } \left( {F_{19} ,\,0.373} \right)$	0.00007	$H_{CE}^{\mu } \left( {F_{20} ,\,0.373} \right)$	0.00052
RD	$H_{CE}^{\mu } \left( {F_{21} ,\,0.373} \right)$	0.00901	$H_{CE}^{\mu } \left( {F_{22} ,\,0.373} \right)$	0.00923	$H_{CE}^{\mu } \left( {F_{23} ,\,0.373} \right)$	0.01402	$H_{CE}^{\mu } \left( {F_{24} ,\,0.373} \right)$	0.01754	$H_{CE}^{\mu } \left( {F_{25} ,\,0.373} \right)$	0.01153
	$H_{CE}^{\mu } \left( {F_{26} ,\,0.373} \right)$	0.01551	$H_{CE}^{\mu } \left( {F_{27} ,\,0.373} \right)$	0.01358	$H_{CE}^{\mu } \left( {F_{28} ,\,0.373} \right)$	0.01596	$H_{CE}^{\mu } \left( {F_{29} ,\,0.373} \right)$	0.01044	$H_{CE}^{\mu } \left( {F_{30} ,\,0.373} \right)$	0.00858

$H_{CE}^{\mu } \left( {F_{1} ,0.3646} \right) = H_{CE}^{\mu } \left( {0.3858,0.3646} \right) = 0.01867,H_{CE}^{\mu } \left( {F_{2} ,0.3646} \right) = H_{CE}^{\mu } \left( {0.4629,0.3646} \right) = 0.01474,...,H_{CE}^{\mu } \left( {F_{30} ,0.3646} \right) = H_{CE}^{\mu } \left( {0.3646,0.3646} \right) = 0.0000$ and so on.

The hyperbolic fuzzy cross entropy measure value set $H_{CE}^{\mu } \left( {A_{1} ,0.3646} \right)$ s between the faulted conditions SC, AD and RD, and healthy condition in pre-defined frequency ranges can be computed employing the resulting Eqs. (12), (13), (14). The results are depicted in Table 9. For instance, we have

**Table 9:** Computation of FCE measure values between the fuzzy sets of artificial winding defects (SC, AD and RD) and healthy condition in pre-defined frequency bands.

Band	Methods	SC	AD	RD	Identified ranking order	Actual ranking order
Low frequency	Our method	**0.16519**	0.00019	0.00013	SC > AD ≈ RD	SC > AD ≈ RD
	Bhandari and Pal	**1.30557**	0.00159	0.00109	SC > AD ≈ RD	SC > AD ≈ RD
	Shiang and Jiang	**0.31837**	0.00040	0.00027	SC > AD ≈ RD	SC > AD ≈ RD
Middle frequency	Our method	0.00043	**0.11621**	0.00035	AD > SC ≈ RD	AD > SC ≈ RD
	Bhandari and Pal	0.00405	**0.99470**	0.00327	AD > SC ≈ RD	AD > SC ≈ RD
	Shiang and Jiang	0.00101	**0.23265**	0.00081	AD > SC ≈ RD	AD > SC ≈ RD
High frequency	Our method	0.00231	0.00241	**0.12540**	RD > AD ≈ SC	RD > AD ≈ SC
	Bhandari and Pal	0.01900	0.01984	**0.99085**	RD > AD ≈ SC	RD > AD ≈ SC
	Shiang and Jiang	0.00469	0.00490	**0.24215**	RD > AD ≈ SC	RD > AD ≈ SC

Significant values are in bold.

*In low frequency band,*

$$H_{CE}^{\mu } \left( {A_{1} ,\,0.3646} \right) = \sum\limits_{j = 1}^{10} {H_{CE}^{\mu } \left( {F_{j} ,\,0.3646} \right)} = 0.16519,^{{}} H_{CE}^{\mu } \left( {A_{2} ,\,0.3646} \right) = \sum\limits_{j = 11}^{20} {H_{CE}^{\mu } \left( {F_{j} ,\,0.3646} \right)} = 0.00019,^{{}} H_{CE}^{\mu } \left( {A_{3} ,\,0.3646} \right) = \sum\limits_{j = 21}^{30} {H_{CE}^{\mu } \left( {F_{j} ,\,0.3646} \right)} = 0.00013.$$

*In middle frequency band,*

$$H_{CE}^{\mu } \left( {A_{1} ,\,0.2947} \right) = \sum\limits_{j = 1}^{10} {H_{CE}^{\mu } \left( {F_{j} ,\,0.2947} \right)} = 0.00043,^{{}} H_{CE}^{\mu } \left( {A_{2} ,\,0.2947} \right) = \sum\limits_{j = 11}^{20} {H_{CE}^{\mu } \left( {F_{j} ,\,0.2947} \right)} = 0.11621,^{{}} H_{CE}^{\mu } \left( {A_{3} ,\,0.2947} \right) = \sum\limits_{j = 21}^{30} {H_{CE}^{\mu } \left( {F_{j} ,\,0.2947} \right)} = 0.00035.$$

*In high frequency band,*

$$H_{CE}^{\mu } \left( {A_{1} ,\,0.373} \right) = \sum\limits_{j = 1}^{10} {H_{CE}^{\mu } \left( {F_{j} ,\,0.373} \right)} = 0.00231,^{{}} H_{CE}^{\mu } \left( {A_{2} ,\,0.373} \right) = \sum\limits_{j = 11}^{20} {H_{CE}^{\mu } \left( {F_{j} ,\,0.373} \right)} = 0.00241,^{{}} H_{CE}^{\mu } \left( {A_{3} ,\,0.373} \right) = \sum\limits_{j = 21}^{30} {H_{CE}^{\mu } \left( {F_{j} ,\,0.3737} \right)} = 0.12540.$$

Next, the Eqs. (12), (13), (14) use to calculate the Bhandari and Pal measure values^23^ between the faulted conditions SC, AD and RD, and healthy conditions. The results are provided in Table 9. For instance, we have:

*In low frequency band*

$$H_{B}^{\mu } \left( {A_{1} ,\,0.3646} \right) = \sum\limits_{j = 1}^{10} {H_{B}^{\mu } \left( {F_{j} ,\,0.3646} \right)} = 1.30557,^{{}} H_{B}^{\mu } \left( {A_{2} ,\,0.3646} \right) = \sum\limits_{j = 11}^{20} {H_{B}^{\mu } \left( {F_{j} ,\,0.3646} \right)} = 0.00159,^{{}} H_{B}^{\mu } \left( {A_{3} ,\,0.3646} \right) = \sum\limits_{j = 21}^{30} {H_{B}^{\mu } \left( {F_{j} ,\,0.3646} \right)} = 0.00109.$$

Similarly, Shiang and Jiang^23^measure values between the faulted conditions SC, AD and RD, and healthy condition can be computed by using resulting Eqs. (18), (19), (20). The results are displayed in Table 9. For instance, we have:

*In low frequency band*

$$H_{S}^{\mu } \left( {A_{1} ,\,0.3646} \right) = \sum\limits_{j = 1}^{10} {H_{S}^{\mu } \left( {F_{j} ,\,0.3646} \right)} = 0.31837,^{{}} H_{S}^{\mu } \left( {A_{2} ,\,0.3646} \right) = \sum\limits_{j = 11}^{20} {H_{S}^{\mu } \left( {F_{j} ,\,0.3646} \right)} = 0.00040,^{{}} H_{S}^{\mu } \left( {A_{3} ,\,0.3646} \right) = \sum\limits_{j = 21}^{30} {H_{S}^{\mu } \left( {F_{j} ,\,0.3646} \right)} = 0.00027.$$

**Figure 6:**
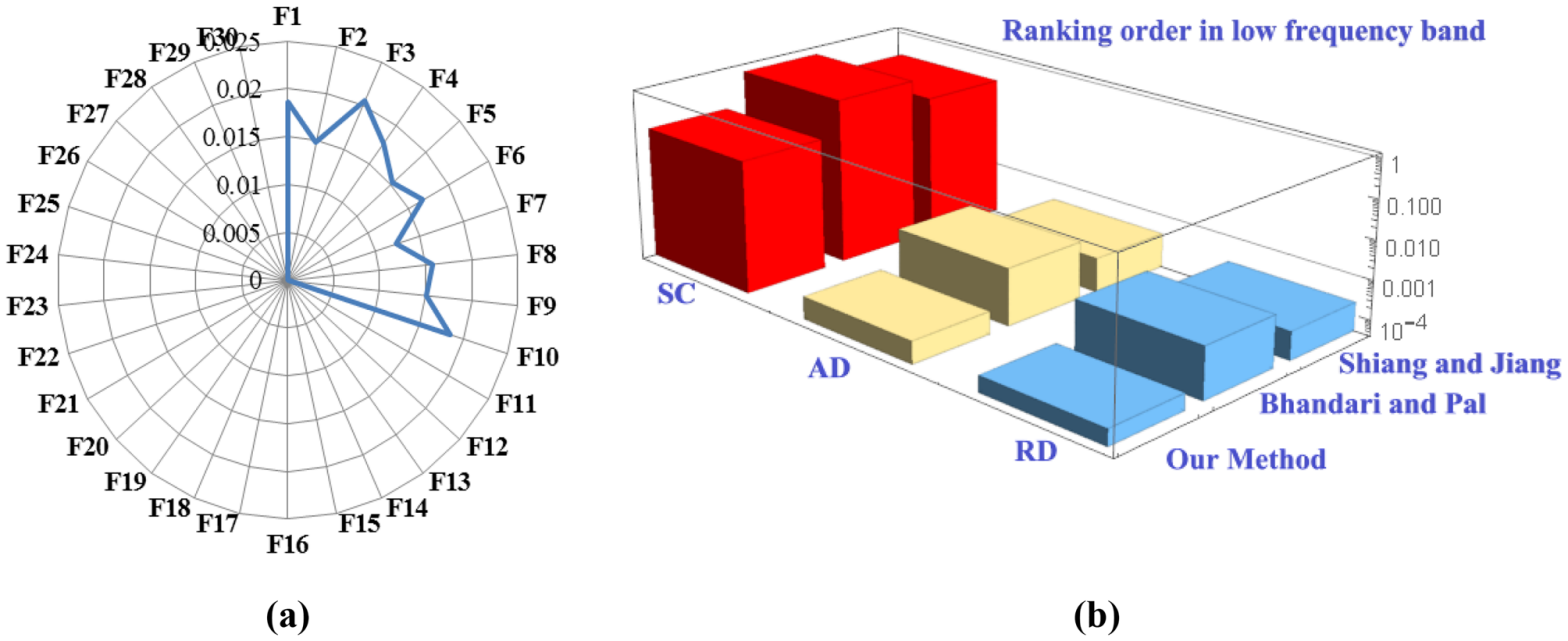
In low frequency range: **(a**) FCE measure values for various faults types **(b)** Comparison sum of measure values of the Shiang and Jiang method^23^, Bhandari and Pal method^23^ and the proposed method for SC, AD, and RD faults.

*Diagnosis result 1.* The highest FCE measure value in low frequency band is 0.16519 (Table 9). Clearly, this value confirms that, in low frequency band, winding fault in the transformer occurs due to the defect in short circuit (faults 1–10). This problem is shown in Fig. 6-a based on the obtained FCE measure value of Table 5. The next smallest FCE measure values are 0.00019 and 0.00013 respectively which correspond to the faulted condition AD and RD. This indicates that, in low frequency band, there is a low possibility of radial and axial deformation in the transformer. Thus, the fault identified classification order, in low frequency range is “SC > AD ≈ RD”. Furthermore, in Table 6, a comparative analysis of the results presented reveals that existing Bhandari and Pal measures^23^, and Shiang and Jiang measures^23^ also return the same fault identified classification order as returned by our proposed FCE measure. This comparison can be seen in Fig. 6b. This justifies the compatibility and reliability of the proposed FCE measure.

**Figure 7:**
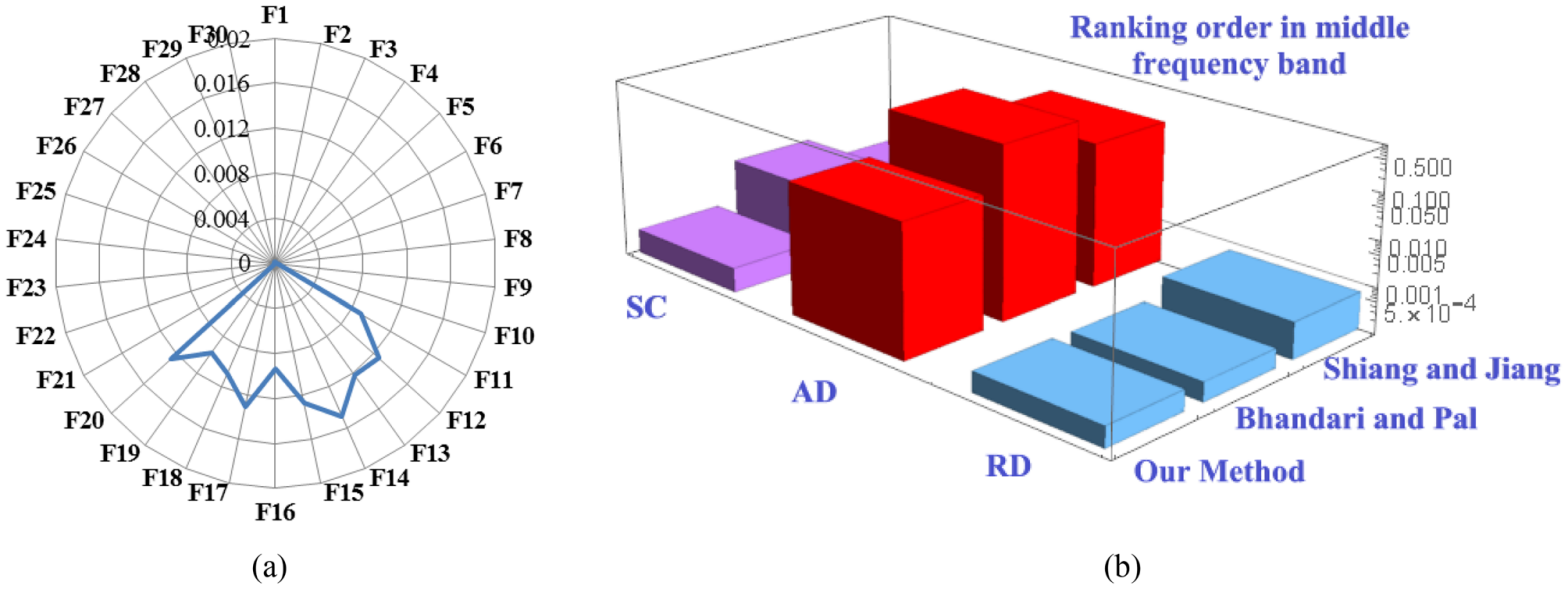
In middle frequency range: (**a**) FCE measure values for various faults types **(b)** Comparison sum of measure values of the Shiang and Jiang method^23^, Bhandari and Pal method^23^and the proposed method for SC, AD, and RD faults.

*Diagnosis result 2.* The highest FCE measure value in middle frequency band is 0.11621 in Table 9. This value indicates that, in middle frequency band, transformer winding fault occurs due to the defect in axial deformation, which is an optimal winding fault selection**.** As it can also be experienced from Fig. 7a for faults 11–20. The next smallest FCE measure values are 0.00043 and 0.00035 respectively which correspond to the faulted condition SC and RD. This indicates that, in middle frequency band, there is a low possibility of short circuit and radial deformation faults in the transformer. Thus, the fault identified classification order, in middle frequency range is “AD > SC ≈ RD”. Furthermore, in Table 6, a comparative analysis of the results presented reveals that existing Bhandari and Pal^23^, and Shiang and Jiang^23^measures also return the same fault identified classification order as returned by our proposed FCE measure. Figure 7-b shows this comparison between the proposed and mentioned methods in the middle frequency. This justifies the compatibility and reliability of the proposed FCE measure.

**Figure 8:**
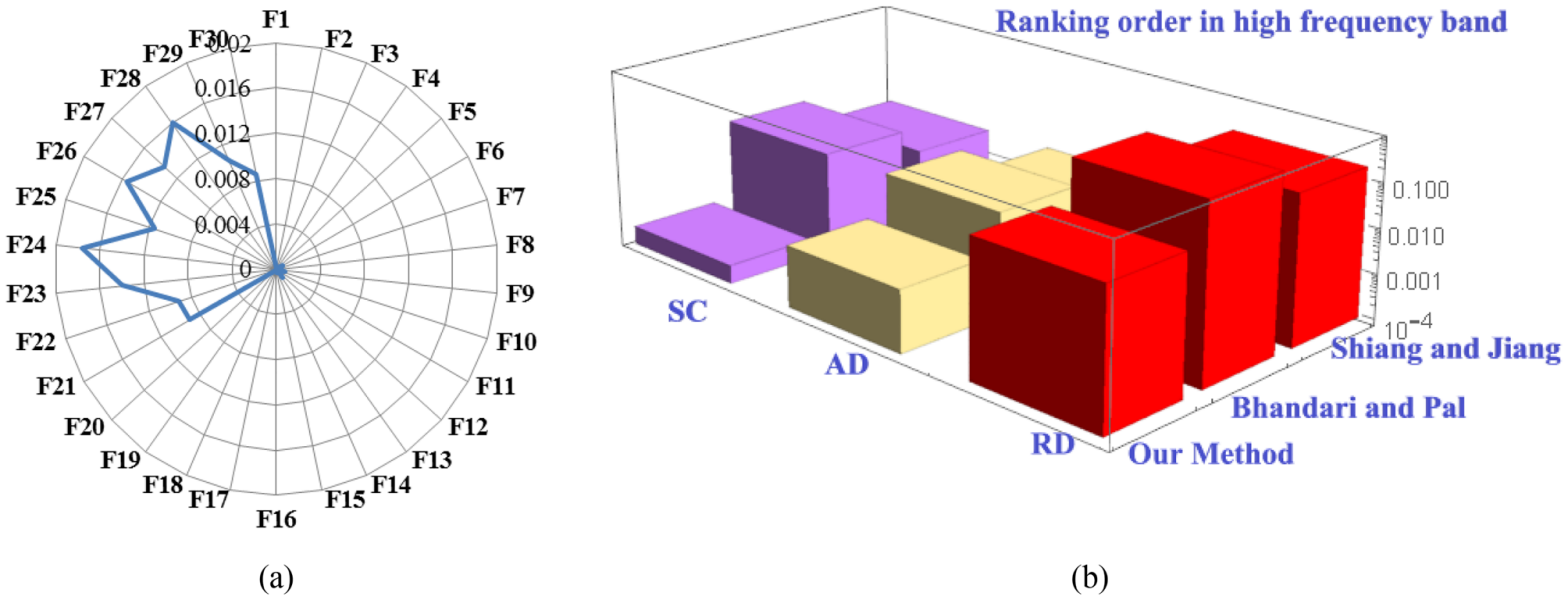
In high frequency range: **(a**) FCE measure values for various faults types **(b)** Comparison sum of measure values of the Shiang and Jiang method^23^, Bhandari and Pal method^23^ and the proposed method.

*Diagnosis result 3.* The highest FCE measure value in high frequency band is 0.12540 in Table 9. This value indicates that, in high frequency band, transformer winding fault occurs due to the defect in radial displacement (Fig. 8a). The next smallest FCE measure values are 0.00231 and 0.00241 respectively which correspond to the faulted condition AD and SC. This indicates that, in high frequency band, there is a low possibility of short circuit and axial deformation faults in the transformer. Thus, the fault identified classification order, in high frequency range is “RD > AD ≈ SC”. This comparison in the high frequency is illustrated in Fig. 8b. This justifies the compatibility and reliability of the proposed FCE measure.

### Discussion

This study successfully applied a new fuzzy cross entropy technique to obtain smart interpretation of FRA results via several winding fault emulating experiments. The normal performance of the transformer is significantly damaged by defects such as AD, SC and RD, so it is necessary to identify and diagnose these defects in time. The windings physical state is changed by these faults types and has a considerable result on the frequency response. Considering that mechanical defects are as effective as the SC faults in the frequency response analysis, it is possible to detect them by the FRA results interpretation via the suggested strategy. For this purpose, a real transformer is used to conduct the essential tests, which include both healthy and faulted circumstances. The measured FRA signatures are classified into three main sub-bands, including > 600, 100–600 and < 100 kHz to better interpret. Then, a new FCE-based approach is offered on the basis of highest and lowest cross entropy measure values. The highest FCE measure values between the fuzzy sets of healthy and faulted circumstances is designated to the detection of occurrence and type of fault. Further examination of the suggested methods results reveals that: (a) In fault occurrence diagnosis, the suggested approach can detect correctly whether the transformer is healthy or faulty, (b) In diagnosing the type of fault, all conditions of the fault are identified correctly, (c) Various fault types of the winding place in various cluster, and there are clear boundaries between them that shows the separability of three types of the winding deformation fault, and (d) The suggested methodology is more accurate and sensitive to mentioned defects than FRA.

## Conclusion

Early diagnosis of problems can prevent a subsequent, catastrophic, electrical failure from occurring in the transformer. A new strategy based on a novel fuzzy cross entropy (FCE) measure for intelligent interpretation of FRA spectrum is presented, practically tested, and assessed in this research. To gather the necessary information, a series of FRA measurements on variously healthy and defective transformer windings must be carried out. FRA traces were studied over three sub-frequency ranges to determine their individual properties and properties of each band. The AD, RD, and SC faults are among those that are being investigated. Winding deformation faults have the property that they can be utilized to identify and classify the primary winding faults. In order to identify efficient characteristics from the produced frequency response analysis results interpretation, the fuzzy sets of healthy and faulted circumstances such as axial, radial and short circuits defect of a transformer. The FCE measure values in the pre-defined low-frequency range are computed as 0.16519, 0.00019 and 0.00013 respectively. All these cross entropy values confirm that the fault identified classification order in the basic sub-range including < 100 kHz is “SC > AD ≈ RD”. This indicates that transformer winding faults in the low frequency band occur due to the defects in short circuits. Furthermore, in low frequency band, there is low possibility of AD and RD winding faults in the transformer. The results obtained through the suggested hyperbolic fuzzy cross entropy-based method have been compared those obtained from the existing fuzzy cross entropy measures. It is revealed that the our proclaimed FCE measure-based distinction and taxonomy of transformer winding faults methodology is compatible and reliable. The proposed approaches' performance is tested and compared by applying the experimental data after feature extraction. The efficiency of the suggested hyperbolic symmetric fuzzy cross entropy is justified by categorizing the transformer faults with the help of existing Bhandari and Pal and Shiang and Jiang asymmetric fuzzy cross entropy measures. A powerful predictive tool can be found in the strategy described here.

### Ethical approval

This paper does not contain any studies with human participants or animals performed by any of the authors.

## Data Availability

The datasets analyzed in the current study are not publicly available due to data protection but are available from the corresponding author on reasonable request.
